# A Facile Strategy to Enhance the Dielectric and Mechanical Properties of MWCNTs/PVDF Composites with the Aid of MMA-co-GMA Copolymer

**DOI:** 10.3390/ma11030347

**Published:** 2018-02-27

**Authors:** Shixin Song, Shan Xia, Shangkun Jiang, Xue Lv, Shulin Sun, Quanming Li

**Affiliations:** 1 Engineering Research Center of Synthetic Resin and Special Fiber, Ministry of Education, Changchun University of Technology, Changchun 130012, China; 1201702002@stu.ccut.edu.cn (S.S.); 1201702004@stu.ccut.edu.cn (S.X.); 20142212@stu.ccut.edu.cn (S.J.); lvxue@ccut.edu.cn (X.L.); 2 Key Laboratory of Automobile Materials, College of Materials Science & Engineering, Jilin University, Changchun 130025, China

**Keywords:** poly(vinylidene fluoride), MMA-co-GMA copolymer, dielectric constant, dielectric loss, dispersion

## Abstract

A facile strategy is adopted to prepare carboxylic functionalized multiwalled carbon nanotube (c-MWCNT) modified high dielectric constant (high-*k*) poly(vinylidene fluoride) (PVDF) composites with the aid of methyl methacrylate-co-glycidyl methacrylate copolymer (MG). The MG is miscible with PVDF and the epoxy groups of the copolymer can react with the carboxylic groups of c-MWCNT, which induce the uniform dispersion of c-MWCNT and a form insulator layer on the surface of c-MWCNT. The c-MWCNTs/MG/PVDF composites with 8 vol % c-MWCNT present excellent dielectric properties with high dielectric constant (~448) and low dielectric loss (~2.36) at the frequency of 1 KHz, the dielectric loss is much lower than the c-MWCNT/PVDF composites without MG. The obvious improvement in dielectric properties ascribes to the existence of MG, which impede the direct contact of c-MWCNTs and PVDF and avoid the formation of conductive network. Therefore, we propose a practical and simple strategy for preparing composites with excellent dielectric properties, which are promising for applications in electronics devices.

## 1. Introduction

With the development of the electronics industry, the fabrication of advanced dielectric composites is highly desirable for their applications. Polymer-based composites with excellent dielectric constant attracted intensive attention over the world, which can be used as embedded capacitors, electric actuators, and energy storage devices [1,2,3,4,5,6,7]. Poly (vinylidene fluoride) (PVDF) and its copolymers have been frequently used as a precursor to prepare dielectric materials due to their high dielectric constant, breakdown strength, thermal stability, and favorable flexibility [8,9,10,11,12,13]. The dielectric constant of PVDF and its copolymers are superior than most of the pure polymers, but are still lower than the electroactive crystals and dielectric ceramics. So, how to enhance the dielectric properties of these materials becomes one important research issue.

In general, dielectric ceramics and conductive fillers are usually applied to improve the dielectric properties of polymers. The dielectric ceramics, such as BaTiO_3_, Zeolite, and ZnO have successfully enhanced the dielectric constant of PVDF and its copolymers in some reports [14,15,16,17,18,19]. However, this method shows some faults such as high concentration addition, more pores, poor flexibility, and processability. As for conductive fillers, carbon nanotube, carbon, graphite nanoplates, and metals were widely used to achieve higher dielectric properties for the polymer composites [20,21,22,23,24,25]. Although many investigations have been performed to increase the compatibility between the polymers and the conductive fillers, the uniform dispersion of the fillers in polymer matrix is still a significant challenge [26,27,28,29]. Furthermore, the poor dispersion of conductive fillers can lead to higher leakage current and result in higher dielectric loss.

Among the conductive fillers, carbon nanotubes (CNTs) have attracted much attention by the researchers. CNTs show large aspect ratio and combines with unique mechanical, thermal and electrical stability, which has been used to improve the dielectric constant of PVDF successfully according to the microcapacitor model [30]. However, the dielectric loss of the composite was usually increased obviously when the conductive paths were formed. In other words, the composites changed into conductive materials, rather than dielectric materials after the formation of conductive network, which restricted the further applications as dielectrics materials. Therefore, improve the dispersion of conductive fillers in the polymer matrix and restrain the dielectric loss of composite becomes a vital problem [31,32,33,34,35,36,37]. Thus, surface modification of the CNTs becomes very important. Furthermore, the change of surface properties also enhances the compatibility between CNTs and polymers. Recently, polypyrrole (PPy) coated multi-walled carbon nanotubes (MWCNTs) were used to blend with polystyrene by Nan and co-workers [34]. The composites showed a high dielectric constant (~44), low dielectric loss (<0.07), and large energy density (4.95 J/cm^3^). Similar research has also been reported by Dang [32]. They compared the dielectric properties of PVDF-based composites with pristine MWCNTs and emeraldine base covered core-shell MWCNTs (MEB) as fillers. The results indicated that MEB/PVDF composites presented a lower loss tangent and a higher dielectric constant than MWNTs/PVDF composites. Amorphous carbon covered MWCNTs were used as fillers to blend with PVDF by Guo [37]. The obtained MWCNT@AC/PVDF composites showed obvious improvement in dielectric constant while the dielectric loss was still very low. Apart from the polymer covered conductive fillers, inorganic particles coated conductive fillers are also employed to restrain the dielectric loss [38,39]. Wu and co-workers prepared TiO_2_ nanorod-decorated CNTs/polystyrene composites [38]. The results indicated that the composite with 17.2 vol % CNTs exhibited a high dielectric constant of 37 and low dielectric loss (~0.11) at 1 KHz. All these strategies can effectively enhance the dielectric properties of polymer composites since the conductive fillers can’t connect directly in the polymer matrix, therefore, the leakage currents and dielectric loss can be reduced. However, most of the treatment process of carbon nanotubes for these reports is often complex and it is difficult to be applied in electronics industry. Therefore, it is necessary and urgent to prepare the excellent dielectric materials by a simple and effective method to meet more industrial demands.

Herein, we report a facile and effective method to prepare high dielectric constant (high-*k*) carboxylic functionalized multiwalled carbon nanotubes (c-MWCNTs) and PVDF nanocomposites with the aid of methyl methacrylate-co-glycidyl methacrylate copolymer (MG). The MG copolymers can cover on the surface of c-MWCNTs due to the chemical reactions between the carboxyl groups of c-MWCNTs and the epoxy groups of MG. The MG serves as insulating layer between the adjacent c-MWCNTs and contributes to the dispersion of c-MWCNTs in the matrix. As a result, it is hard to form a conductive path between the c-MWCNTs. Moreover, the leakage current and resultant dielectric loss can be suppressed effectively. The composites that were prepared with this method exhibit high dielectric constant and low dielectric loss. This research provides a flexible strategy to fabricate composites with excellent dielectric performance and the similar strategy can be used for other dielectric polymer composites.

## 2. Materials and Methods

### 2.1. Materials

c-MWCNTs were purchased from Chengdu Institute of Organic Chemistry, Chinese Academy of Science (Chengdu, China). The outer and inner diameters of c-MWCNTs are 10~20 nm and 5~10 nm, respectively. The length of a single c-MWCNT is about 10~30 μm and the -COOH content is 2 wt %. PVDF (Solef 6010, Tavaux, France) was commercially purchased from Dongguan Wenjin New Materials Co. Ltd. (Dongguan, China). The MG was prepared in our lab and the preparation process can be found from the reference [40].

### 2.2. Preparation of c-MWCNTs/MG Composites

In order to allow for the chemical reaction between c-MWCNTs and MG, 5 g MG was dissolved into 150 mL DMAc and then 0.15 g c-MWCNTs were added under ultrasonication for 20 min. Subsequently, the mixture was separated and dried in a vacuum oven, and the result composites were hot pressed for 10 min at the temperature of 200 °C. Then, the composites were dissolved into DMAc again and were centrifuged to remove the MG without reaction. The resulted composites were used for other tests.

### 2.3. Preparation of c-MWCNTs/PVDF and c-MWCNTs/MG/PVDF Composites

The c-MWCNTs/MG/PVDF composites were prepared by melt blending method. The c-MWCNTs, MG and PVDF were dried in a vacuum oven at 80 °C for 24 h to remove the moisture before proceeding. The blending was carried out in a Thermo Haake internal mixer (Thermo Scientific, Karlsruhe, Germany). The temperature was set at 200 °C with a rotation speed of 60 rpm and the mixing time was 5 min. As for all of the c-MWCNTs/MG/PVDF composites, the weight ratio of MG and PVDF was 1:9. The volume fractions of c-MWCNTs in total composites were 0.8, 2.4, 4.0, 5.6, 7.2, 8.0 and 9.6%, and the corresponding composites were defined as PMG0.8, PMG2.4, PMG4.0, PMG5.6, PMG7.2, PMG8.0, and PMG9.6, respectively. In addition, the MG/PVDF composite without c-MWCNTs was also prepared and named as PMG0. After blending, the samples with different compositions were obtained by hot press molding for 3 min at 200 °C and cold press molding for 3 min at room temperature. In order to investigate the effect of MG on the properties of composite, the composites without MG (c-MWCNTs/PVDF) were prepared with the similar procedure. The volume fractions of c-MWCNTs in c-MWCNTs/PVDF composites were 0.4, 0.8, 1.2, 1.6, 2.0, and 2.4%, and the resulting composites, named as 0.4CNT, 0.8CNT, 1.2CNT, 1.6CNT, 2.0CNT, and 2.4CNT, respectively.

### 2.4. FTIR Tests

Fourier transform infrared spectroscopy (FTIR) was performed on a Thermo Nicolet Avatar-360 (Thermo Nicolet, Gilroy, CA, USA) to confirm the reaction between c-MWCNTs and MMA-co-GMA copolymer. FTIR measurement was recorded from 650 cm^−1^ to 4000 cm^−1^ with a resolution of 4 cm^−1^ and 32 scans.

### 2.5. TGA Test

Thermo gravimetric analysis (TGA) was performed on a Pyris-1 thermal analyzer (Perkin-Elmer, Waltham, MA, USA) with temperature range from 30 to 600 °C in a nitrogen atmosphere.

### 2.6. Mechanical Tests

The tensile tests were performed with an Instron-3365 tensile tester (Instron, Boston, MA, USA), according to ASTM D638 at a crosshead speed of 50 mm/min at 23 °C.

### 2.7. Morphology Observation

Scanning Electron Microscopy (SEM) micrographs were used to observe the dispersion of c-MWCNTs in PVDF using a JSM6510 scanning electron microscope (JEOL, Tokyo, Japan). Before testing, all of the samples were coated with a gold layer for SEM observation with an operation voltage of 10 kV.

### 2.8. Dielectric Properties

Dielectric measurements were performed on LCR Meter (E4980A, Keysight Technologies, Santa Clara, CA, USA), with the frequency ranging from 20 Hz to 2 MHz at room temperature. The samples with a diameter of 60 mm and thickness of 1 mm were put into the two electrodes to be measured. Thus, the dielectric constant and dielectric loss were tested.

### 2.9. Viscosity Test

Rheological measurements were performed on a rheometer (AR2000EX, TA Instruments-Waters LLC, New Castle, PA, USA) at 200 °C and the oscillatory frequency sweep ranged from 0.1 to 100 rad/s.

## 3. Results and Discussion

### 3.1. Compatibilization Mechanisms

**Figure 1:**
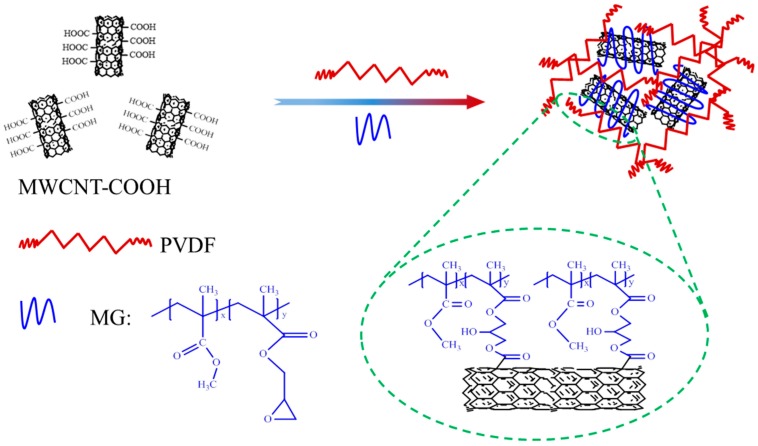
Reactive mechanism between methacrylate copolymer (MG) copolymers and carboxylic functionalized multiwalled carbon nanotube (c-MWCNTs).

**Figure 2:**
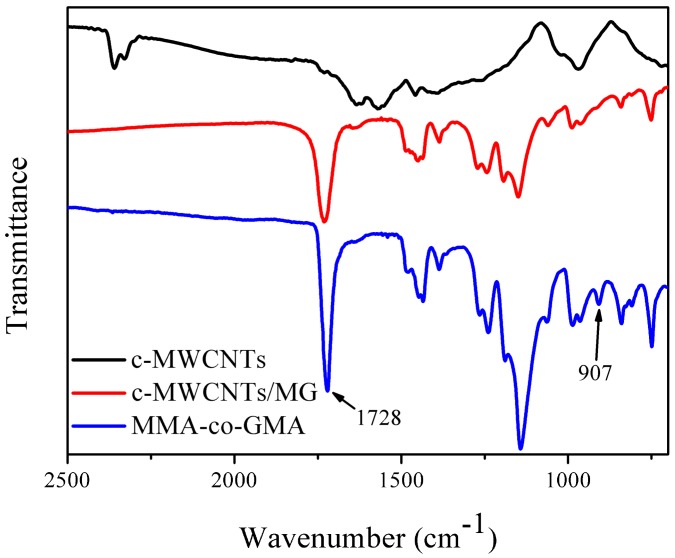
Fourier transform infrared spectroscopy (FTIR) spectrum of pristine c-MWCNTs, MG copolymers, and c-MWCNTs/MG composites.

The MMA-co-GMA copolymers were fabricated via a continuous solution polymerization method. The compatibility between MG and PVDF has been reported in the previous research [40]. Figure 1 displays the reactive mechanism between the epoxy groups of MG and the carboxyl groups of c-MWCNTs, which can be testified by the following tests. Figure 2 shows the FTIR spectrum and the dispersion status of pristine c-MWCNs, MG copolymer and as-prepared c-MWCNTs/MG composite. MG copolymers show the characteristic peaks at 1728 cm^−1^ and 907 cm^−1^, which belong to the carbonyl and epoxy groups, respectively. As for the separated c-MWCNTs/MG composite, the carbonyl peak at 1728 cm^−1^ still exists, however, the peak at 907 cm^−1^ disappears in the spectrum of c-MWCNTs/MG copolymers. The change of the FTIR result indicates that the epoxy groups of MG copolymers have reacted with the carboxyl groups of the c-MWCNT [41]. Therefore, FTIR results indicate that MG copolymers have been grafted on the surface of c-MWCNT successfully.

**Figure 3:**
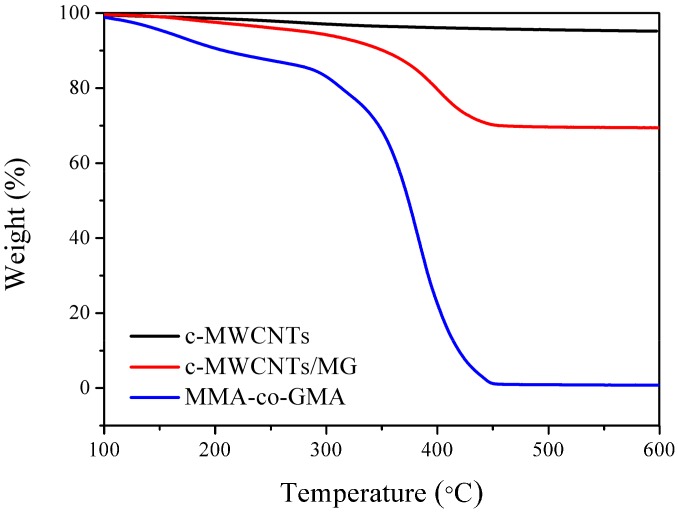
TGA curves of pristine c-MWCNTs, pure MG and c-MWCNTs/MG composites.

The TGA curves of pristine c-MWCNTs, MG copolymer, and c-MWCNT/MG composites are shown in Figure 3. It can be observed that the weight loss of pristine c-MWCNTs at 600 °C is about 4.7 wt % due to the thermal oxidation and decomposition of carboxyl and hydroxyl groups on the c-MWCNTs at high temperature. The MG copolymer almost decomposes totally at 600 °C. In comparison, the weight loss of c-MWCNT/MG composites is about 30.4 wt % at 600 °C. The weight loss contributed to the decomposition of MG copolymers grafted on the surface of c-MWCNTs, which shows much higher decomposition temperature than the pure MG copolymers. Therefore, the TGA results further indicate that MG copolymers have grafted on the c-MWCNTs surface successfully.

**Figure 4:**
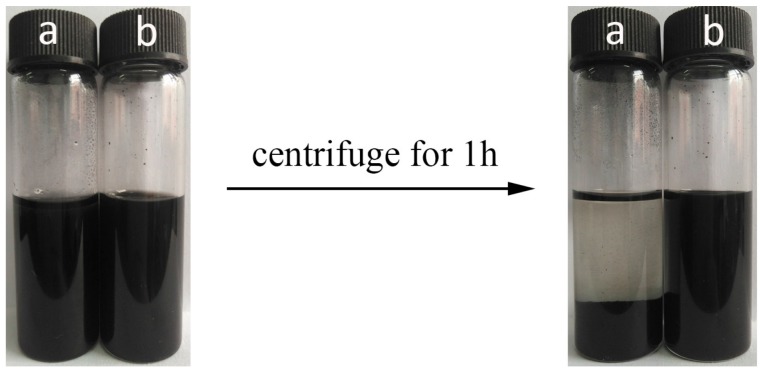
Dispersion photographs of (**a**) pristine c-MWCNTs and (**b**) c-MWCNTs/MG composite in DMAc before and after centrifugation.

Figure 4 exhibits the dispersion of the pristine c-MWCNTs and the c-MWCNTs/MG composite in solvent before and after centrifugation. As shown in Figure 4, the pristine c-MWCNTs precipitate completely in DMAc, while c-MWCNT/MG composites still disperse stably in the solution after centrifugation. The enhanced dispersion stability of c-MWCNTs/MG composites in DMAc can be attributed to the grafting of MG copolymers on the c-MWCNTs surface, which improve the compatibility between c-MWCNTs and the solvent. Therefore, dispersion experiment indicates that MG copolymers can react with carboxylic functionalized c-MWCNTs, and the resultant c-MWCNTs/MG shows good dispersion in solvent after centrifugation.

**Figure 5:**
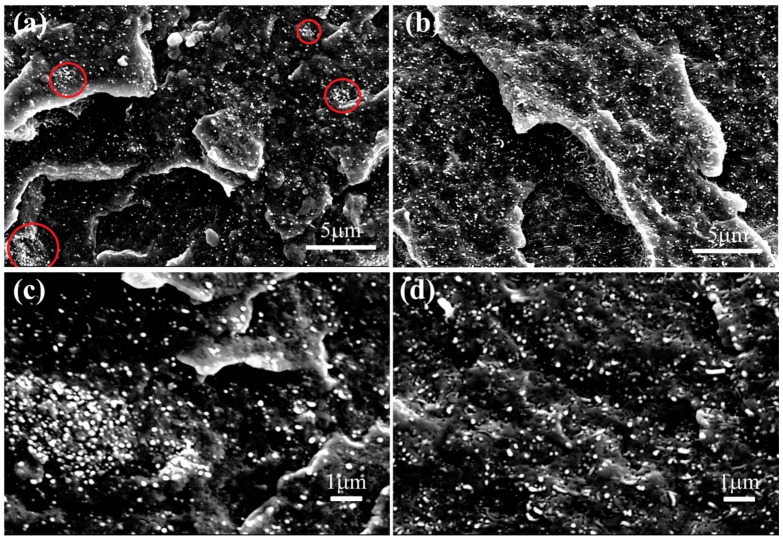
SEM images of the composite filled with 2.4 vol % c-MWCNTs. (**a**) c-MWCNT/PVDF composite with 5000 magnification; (**b**) c-MWCNT/MG/PVDF composite with 5000 magnification; (**c**) c-MWCNT/PVDF composite with 10,000 magnification; and, (**d**) c-MWCNT/MG/PVDF composite with 10,000 magnification.

**Figure 6:**
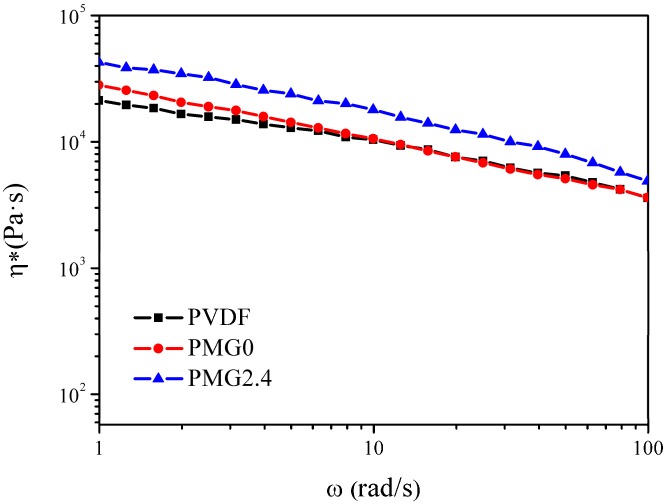
Complex viscosity (η*) versus frequency for the neat PVDF, PMG0 and c-MWCNTs/MG/PVDF with 2.4 vol % c-MWCNTs.

Scanning electron microscope (SEM) was used to observe the dispersion of c-MWCNTs in the matrix. Figure 5 shows the SEM images of c-MWCNT/PVDF and c-MWCNT/MG/PVDF composites. It can be clearly found that partial c-MWCNTs aggregate together in the PVDF matrix due to the poor compatibility between the c-MWCNTs and PVDF (Figure 5a, the aggregated particles are marked with red circles.). Furthermore, more obvious aggregation can be observed in Figure 5c. However, as for the c-MWCNT/MG/PVDF composites, no aggregations occurred, as shown in Figure 5b,d. This is mainly due to the compatibilization effect of MG copolymers on the c-MWCNTs surface. MG copolymers serve as a compatibilizer, and enhance the compatibility between the c-MWCNTs and the PVDF matrix, leading to uniform dispersion of the c-MWCNTs in the PVDF matrix. In addition, the viscosity of polymer matrix can also affect the dispersion of c-MWCNTs. The complex viscosity (η*) of pure PVDF is slightly lower than that of PVDF/MG blend (PMG0) at low frequency and there is no obvious difference at high frequency, as shown in Figure 6. When c-MWCNT was added into the blend, the viscosity of the composite further increased. Therefore, the increment of viscosity for PVDF due to MG is beneficial to the uniform dispersion of c-MWCNTs in the composites. The SEM and viscosity test results prove that the compatibilization effect and the viscosity improvement of PVDF matrix due to MG addition promote the uniform dispersion of c-MWCNTs appropriately.

### 3.2. Dielectric Properties

**Figure 7:**
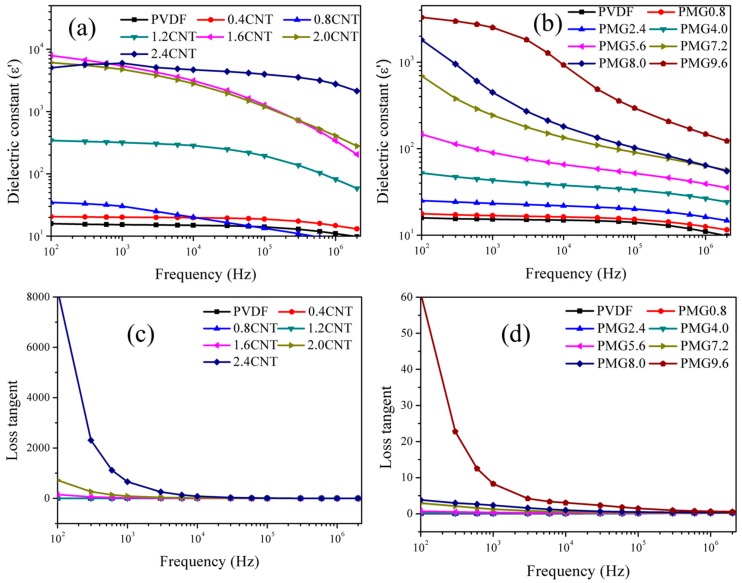
Frequency dependent of c-MWCNTs/PVDF and c-MWCNTs/MG/PVDF composites with different c-MWCNTs contents: (**a**,**b**) dielectric constant, (**c**,**d**) loss tangent.

In conductive particles filled composites, the percolation threshold (fc) plays an important role in electric conductivity and dielectric properties. Percolation is not merely the significant changes of structure, but leads to dramatic changes in physical properties of composites when the content of conductive fillers approaches fc. Therefore, percolation is considered as a powerful tool to predict the physical properties of heterogeneous composites. For MWCNTs filled composites, there are significant differences in dielectric parameters when the volume fraction of MWCNTs is below, near, and above the percolation threshold. When the MWCNTs content is below the percolation threshold, according to the microcapacitor model [3,24,32], the number of microcapacitors is inadequate and the microcapacitors behave very low capacitance which results in a slight increase of dielectric constant. Once the volume fraction of MWCNTs is near percolation threshold, the dielectric constant of the composites improves significantly due to the increment of microcapacitor number and the improvement of capacitance. When the MWCNTs content is beyond the percolation threshold, the conductive paths are formed and the composite turns into conducting material rather than dielectrics. In this condition, the dielectric loss increases significantly due to the formation of leakage current. In the present research, the percolation threshold of the composites can be calculated by the following equation:

$$\varepsilon_{\mathrm{eff}}\;\propto \varepsilon_{\mathrm{PVDF}}{\left(f_{c}-f\right)}^{-s}\;\mathrm{for}\;f<f_{c}$$

where ε_eff_ is the dielectric constant of composite, ε_PVDF_ is the dielectric constant of PVDF, *f_c_* is the percolation threshold, *f* is the volume fraction of fillers, *s* is the critical exponent. The best fits of electrical data, according to the power law gives that *f_c_* = 1.37 vol % for c-MWCNTs/PVDF composites and *f_c_* = 7.32 vol % for c-MWCNTs/MG/PVDF composites. The increase of the percolation threshold is mainly due to the presence of MMA-co-GMA copolymers, which prevent the direct contact of c-MWCNTs and increase the difficulty to form conductive path in c-MWCNTs/MG/PVDF blends. Figure 7 shows the frequency dependence of the dielectric parameters of the c-MWCNTs/PVDF and c-MWCNTs/MG/PVDF composites at room temperature. As shown in Figure 7a,b, when the filler contents are below the percolation threshold, the dielectric constant of c-MWCNTs/PVDF and c-MWCNTs/MG/PVDF composites decreases slowly with the increase of frequency. Both composites exhibit high dielectric constant at 100 Hz, which is due to the charge carriers accumulation at the internal interfaces under an external electric field, according to the Maxwell-Wagner-Sillars (MWS) effect, because of the abundant nomadic electrons in MWCNT and the formation of numbers of interfaces between the MWCNTs and PVDF. While, as the filler contents increase, the dielectric constant of both composites exhibits high frequency dependence. When the filler concentrations exceed the percolation threshold, it can be found that the dielectric constant decreases gradually with the frequency until 10^5^ Hz, suggesting an obvious dielectric relaxation behavior at high frequency. Therefore, the behavior of frequency dependence indicates that interfacial polarization is the main mechanism in capacitance of these microcapacitors. In addition to this, as shown in Figure 7c,d, it can be found that the loss tangent of c-MWCNTs/PVDF and c-MWCNTs/MG/PVDF composites decreases with the increase of frequency. Furthermore, the loss tangent of the both composites exhibits high frequency dependence, which is consistent with the dielectric constant of the composites.

**Figure 8:**
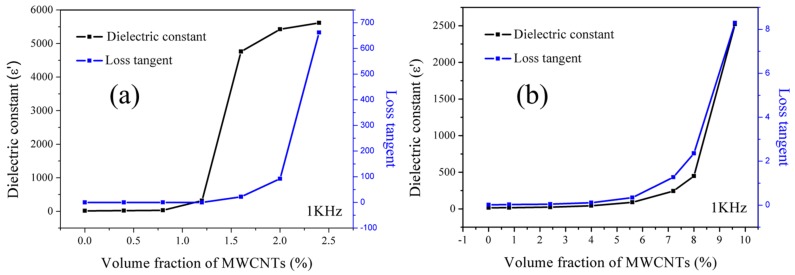
Dielectric parameters of (**a**) c-MWCNTs/PVDF and (**b**) c-MWCNTs/MG/PVDF composites with different c-MWCNTs contents measured at room temperature with the frequency of 1 kHz.

Figure 8 shows the dielectric constant and loss tangent of the two composites with different conductive filler concentrations, measured at the frequency of 1 KHz and room temperature. Obviously, it can be observed that the dielectric constant of both the composites increases with the addition of fillers, which can be explained by the microcapacitor theory. In polymer-based composites, the conductive fillers serve as microcapacitor, which can contribute to the improvement of dielectric constant. Furthermore, the dielectric constant is closely related to the amount of microcapacitor in composites. When the filler content is lower than percolation threshold, the number of microcapacitors in composites is insufficient and the dielectric constant increases slightly. With the increase of filler contents, especially when the filler content exceeds to the percolation threshold, the amount of microcapacitors increases a lot, which results in a sharp improvement of dielectric constant. In this case, the conductive network and leakage current can also be formed in the composites. Inevitably, the loss tangent of the composites increases greatly due to the resultant leakage loss. Therefore, the composites cannot be used as dielectric materials due to the high loss tangent and conductive characteristics. On the other hand, for the composites of c-MWCNTs/PVDF with 2.4 vol % c-MWCNTs contents, the dielectric constant can reach about 5616, which is two times higher than the dielectric constant of c-MWCNTs/MG/PVDF composites at the c-MWCNTs content of 9.6 vol % (~2521). In addition to this, the c-MWCNTs/PVDF composites exhibit higher dielectric loss (~662), which is far surpassed than that of c-MWCNTs/MG/PVDF composites (~8.29). The obvious difference between the two composites is mainly due to the addition of MMA-co-GMA copolymers, which can contribute to the dispersion of c-MWCNTs in the matrix and hinder the direct contact of c-MWCNTs. As a result, the loss tangent resulting from the leakage current in composites can be effectively reduced. Furthermore, even the content of c-MWCNTs in the c-MWCNTs/MG/PVDF composites is much more than that of c-MWCNTs/PVDF composites; the loss tangent of c-MWCNTs/MG/PVDF composites still remains a low value. Therefore, the c-MWCNTs/MG/PVDF composites exhibit a comparatively high dielectric constant and low loss tangent.

Table 1 shows the comparative dielectric parameters of PVDF-based composites reported in the corresponding literatures with our research. NH_2_-CuPc grafted multiwalled carbon nanotubes were used by Zhang to improve the dielectric properties of PVDF [42]. It can be found that the composites with 10 wt % MWCNTs-CuPc have a dielectric constant of 45 and low dielectric loss of 0.15 at the frequency of 1 KHz. Sun prepared the POSS-coated MWCNTs to compound with PVDF [43]. The dielectric constant and dielectric loss of 5 wt % POS@CNT-B5/PVDF composite were about 98 and 0.53 at the frequency of 1 KHz, respectively. However, at the same frequency, the POS@CNT-B5/PVDF composites exhibited giant dielectric loss (~155), while the dielectric constant did not increase a lot (~146) when the filler contents reached to 6 wt %. Surface-functionalized MWNTs with emeraldine base (MEB) was prepared by Zhou to tailor the dielectric properties of PVDF [32]. The resulted MEB/PVDF composites with 8.3 vol % MWCNTs exhibited high dielectric permittivity and a low loss tangent at the frequency of 1 KHz, which are about 260 and 1.3, respectively. Trifluorobromobenzene treated MWCNTs were blended with PVDF by Dang [24]. It can be found that the dielectric constant of the composites with 8 vol % MWCNTs can reach about 550 while the dielectric loss is only about 2.2 at the frequency of 10^3^ Hz. All of the composites that were prepared by these methods have a certain improvement in dielectric properties. However, it should be noted that all these works require complicated pretreatment of MWCNTs, which is time-consuming and inefficient in industry. As for the dielectric parameters in the present paper, the dielectric constant and loss tangent are about 448 and 2.35 for c-MWCNTs/MG/PVDF composite with 8 vol % c-MWCNTs measured at the frequency of 1 KHz. Even though the loss tangent is slightly higher than that of the references, simple, and flexible preparation process is advantageous for our method. In addition, the prepared composites exhibit high dielectric constant.

### 3.3. Electric Properties

**Figure 9:**
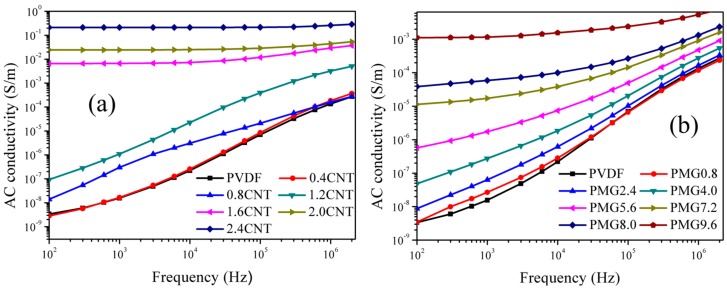
Frequency dependent of the ac conductivity of (**a**) c-MWCNTs/PVDF and (**b**) c-MWCNTs/MG/PVDF composites with different c-MWCNTs contents.

Electron transport mechanism in conductive particles filled composites has been reported in many papers [44,45,46]. Brosseau and co-workers analyzed the ac conductivity of reduced graphene oxide/epoxy polymer composites, and they found that the electrical properties were strongly influenced by graphene oxide content [45]. They proposed that the electron hopping was the ac transport mechanism when the graphene oxide content was below the percolation threshold. Once the volume fraction of graphene oxide was near and above the percolation threshold, the ac conduction originated from both electron tunneling and capacitive paths among the reduced graphene oxide nanoparticles in the polymer bulk. Gu et al. reported that direct contact of conductive particles and electrons transmit through electron tunnels were the two mechanisms for electrical transport of carbon nanotubes/epoxy composites [46]. The resultant current is named as leakage current and tunneling current, respectively. Furthermore, the leakage current can contribute more to the conductivity of composites than tunneling current. Figure 9 shows the frequency dependence of ac conductivity for c-MWCNTs/PVDF and c-MWCNTs/MG/PVDF composites with different filler contents at room temperature. It is found that the ac conductivities of both the composites are closely related to the contents of filler and frequency. At low filler content, the ac conductivities increase slowly with the increase of filler contents for both of the composites. Furthermore, the c-MWCNTs loading are not sufficient to form a conductive network when the content of filler is small, hence the tunneling current is the main conductive mechanism. In this case, the ac conductivity of composites exhibits an almost linear relationship with frequency at low frequency. However, for c-MWCNTs/PVDF composites, when the volume fraction of c-MWCNTs is more than 1.2 vol %, the ac conductivity increases dramatically, and which can reach to 0.213 S/cm at 1000 Hz for 2.4 vol % c-MWCNTs. When the filler content is more than percolation threshold, the c-MWCNTs contact with each other and form a conductive network, in this condition, the leakage current is the main factors for the conductivity increase. Moreover, the ac conductivity is no longer dependent of frequency due to the formation of conductive network at low-frequency, which is consistent with the typical percolation phenomenon. For the composites of c-MWCNTs/MG/PVDF, the ac conductivity increases significantly when the filler loading exceeds 7.2 vol % and the percolation threshold enhances about six times of magnitude than that of c-MWCNTs/PVDF composites, however, the ac conductivity is much lower than that of c-MWCNTs/PVDF composites. This can be explained that the MMA-co-GMA copolymer can act as insulating layer, which not only contributes to the dispersion, but also prevents the direct contact of c-MWCNTs in matrix. Therefore, it is disadvantageous to the formation of a conductive network. In this situation, electrons can only be transmitted through electron tunnels between adjacent carbon nanotubes; hence, tunneling currents become the dominant mechanism.

### 3.4. Mechanical Properties

**Figure 10:**
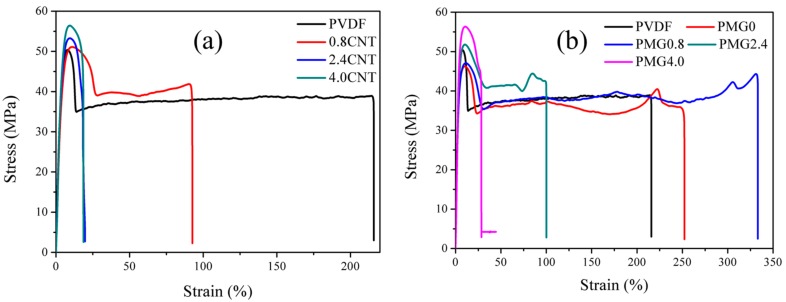
Stress-strain curves of (**a**) PVDF and c-MWCNTs/PVDF composites with 0.8 vol %, 2.4 vol % and 4.0 vol % c-MWCNTs; (**b**) PVDF, PMG0 and c-MWCNTs/MG/PVDF composites with 0.8 vol %, 2.4 vol % and 4.0 vol % c-MWCNTs.

In Figure 10, the stress-strain curves of the c-MWCNTs/PVDF composites and c-MWCNTs/MG/PVDF composites were presented. It is evident that the addition of c-MWVNTs can improve the tensile strength of PVDF, as shown in Figure 10a. As for the MWCNTs/MG/PVDF composites, it can be found that the tensile strength of PMG0 was about 46.2 MPa, which is lower than PVDF of 50.3 MPa. This is mainly due to the inhibited crystallization of PVDF with the existence of MMA-co-GMA copolymers. On the other hand, with the addition of c-MWCNTs, the tensile strength of c-MWCNTs/MG/PVDF composite is increased. The tensile strength of PMG4.0 blend can reach to 56.3 MPa. These results indicate that MWCNTs can effectively enhance the tensile strength of the composites. Furthermore, the elongation at break of the PVDF blends decreases compared with pure PVDF. The elongation at break of 0.8CNT blend can reach about 93%, however, the elongation at break of PMG0.8 composite can reach about 333% in Figure 10b, which is more than three times higher than that of 0.8CNT composite. Similar conditions are also appeared in other components. For example, the elongation at break of PMG2.4 is about 100% when compared with 2.4CNT of 20%. The obvious enhancement of elongation at break of MWCNTs/MG/PVDF is mainly due to the existence of MMA-co-GMA copolymers, which can not only contribute to the dispersion of MWCNTs but also enhance the interfacial strength between MWCNTs and PVDF matrix.

## 4. Conclusions

In conclusion, we have presented a flexible method to prepare c-MWCNTs/MG/PVDF composites by using MMA-co-GMA copolymers as compatibilizer between c-MWCNTs and PVDF. The resultant c-MWCNTs/MG/PVDF composites exhibit excellent dielectric properties when compared with the composites of c-MWCNTs/PVDF. The improvement in dielectric properties of c-MWCNTs/MG/PVDF composites attribute to the introduction of MMA-co-GMA copolymers, which not only contribute to the uniform dispersion of c-MWCNTs in PVDF, but can also serve as a barrier layer and hinder the direct contact of c-MWCNTs. Besides, the dielectric properties of composites can be tailored by adjusting the content of MMA-co-GMA copolymers to meet more demands. Among the method to fabricate dielectric composites, the implement process is also very important. Previous researches usually need the pretreatment of MWCNTs, which is complex and restrict their further applications in industry. However, in the present research, the whole process does not require complex surface pretreatment of carbon nanotubes and the resultant composites also exhibit enhanced properties. Therefore, this paper provides a practical and simple method for preparing PVDF composites with excellent dielectric properties and the similar strategy can be extended to prepare other dielectric materials.

## Figures and Tables

**Table 1:** Dielectric parameters for our research compared with the corresponding literatures.

Designation Used Here	Filler Content	Frequency	Dielectric Constant	Dielectric Loss
MWCNTs-CuPc (ref. [42])	10 wt %	1 kHz	45	0.15
POS@CNTB5 (ref. [43])	5.0 wt %	1 kHz	98	0.53
MEB (ref. [32])	8.3 vol %	1 kHz	260	1.30
TEP-MWCNT (ref. [24])	8.0 vol %	1 kHz	550	2.20
MG/MWCNT (our research)	8.0 vol %	1 kHz	448	2.35
